# 6,6′-Dieth­oxy-2,2′-[4,5-dimethyl-*o*-phenyl­enebis(nitrilo­methyl­idyne)]diphenol–ethanol–water (1/1/1)

**DOI:** 10.1107/S1600536809008903

**Published:** 2009-03-14

**Authors:** Hadi Kargar, Reza Kia, Arezoo Jamshidvand, Hoong-Kun Fun

**Affiliations:** a Department of Chemistry, School of Science, Payame Noor University (PNU), Ardakan, Yazd, Iran; b X-ray Crystallography Unit, School of Physics, Universiti Sains Malaysia, 11800 USM, Penang, Malaysia

## Abstract

The title bis-Schiff base compound, C_26_H_28_N_2_O_4_·C_2_H_6_O·H_2_O, crystallizes as an ethanol and water solvate. Strong intra­molecular O—H⋯N hydrogen bonds generate *S*(6) ring motifs. The water H atoms form bifurcated O—H⋯(O,O) inter­molecular hydrogen bonds with the O atoms of the hydroxyl and eth­oxy groups with *R*_1_^2^(5) ring motifs, which may, in part, influence the mol­ecular configuration. The dihedral angles between the central benzene ring and the two outer benzene rings of the Schiff base mol­ecule are 5.64 (8) and 44.78 (9)°. The crystal structure is further stabilized by inter­molecular C—H⋯O and π–π inter­actions [centroid–centroid distances = 3.6139 (11)–3.7993 (11) Å].

## Related literature

For bond-length data, see: Allen *et al.* (1987 ▶). For hydrogen-bond motifs, see: Bernstein *et al.* (1995 ▶). For related structures, see, for example: Cakir *et al.* (2002 ▶); Eltayeb *et al.* (2007*a* ▶,*b* ▶); Karabıyık *et al.* (2007 ▶); Fun & Kia (2008 ▶); Fun, Kargar & Kia (2008 ▶); Fun, Kia & Kargar (2008 ▶). For applications of Schiff base ligands, see, for example: Hajioudis *et al.* (1987 ▶); Granovski *et al.* (1993 ▶); Dao *et al.* (2000 ▶); Shahrokhian *et al.* (2000 ▶); Eltayeb & Ahmed (2005**a* ▶,b* ▶); Fakhari *et al.* (2005 ▶); Karthikeyan *et al.* (2006 ▶); Sriram *et al.* (2006 ▶). For the stability of the temperature controller used for the data collection, see: Cosier & Glazer (1986 ▶).
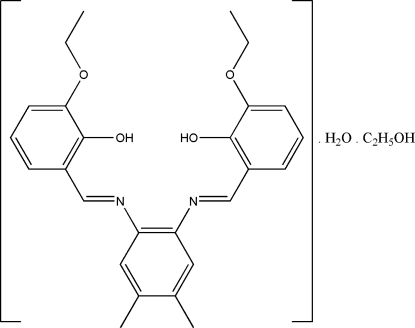


## Experimental

### 

#### Crystal data

C_26_H_28_N_2_O_4_·C_2_H_6_O·H_2_O
*M* *_r_* = 496.59
Monoclinic, 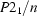

*a* = 9.5095 (5) Å
*b* = 25.6633 (12) Å
*c* = 10.7766 (5) Å
β = 99.177 (2)°
*V* = 2596.3 (2) Å^3^
*Z* = 4
Mo *K*α radiation
μ = 0.09 mm^−1^
*T* = 100 K
0.45 × 0.12 × 0.02 mm

#### Data collection

Bruker SMART APEXII CCD area-detector diffractometer
Absorption correction: multi-scan (**SADABS**; Bruker, 2005 ▶) *T*_min_ = 0.961, *T*_max_ = 0.999
32813 measured reflections
7595 independent reflections
4303 reflections with *I* > 2σ(*I*)
*R*_int_ = 0.068

#### Refinement

*R*[*F* ^2^ > 2σ(*F* ^2^)] = 0.066
*wR*(*F* ^2^) = 0.160
*S* = 1.03
7595 reflections
341 parameters
H atoms treated by a mixture of independent and constrained refinement
Δρ_max_ = 0.38 e Å^−3^
Δρ_min_ = −0.51 e Å^−3^

### 

Data collection: *APEX2* (Bruker, 2005 ▶); cell refinement: *SAINT* (Bruker, 2005 ▶); data reduction: *SAINT*; program(s) used to solve structure: *SHELXTL* (Sheldrick, 2008 ▶); program(s) used to refine structure: *SHELXTL*; molecular graphics: *SHELXTL*; software used to prepare material for publication: *SHELXTL* and *PLATON* (Spek, 2009 ▶).

## Supplementary Material

Crystal structure: contains datablocks global, I. DOI: 10.1107/S1600536809008903/lh2786sup1.cif

Structure factors: contains datablocks I. DOI: 10.1107/S1600536809008903/lh2786Isup2.hkl

Additional supplementary materials:  crystallographic information; 3D view; checkCIF report

## Figures and Tables

**Table 1:** Hydrogen-bond geometry (Å, °)

*D*—H⋯*A*	*D*—H	H⋯*A*	*D*⋯*A*	*D*—H⋯*A*
O1—H1⋯N1	0.84	1.84	2.584 (2)	146
O2—H2⋯N2	0.84	1.87	2.609 (2)	146
O1*W*—H1*W*1⋯O2^i^	0.86 (3)	2.24 (3)	2.947 (2)	139 (3)
O1*W*—H1*W*1⋯O4^i^	0.86 (3)	2.28 (3)	3.018 (2)	145 (3)
O1*W*—H2*W*1⋯O1^i^	0.78 (3)	2.30 (3)	3.061 (2)	166 (3)
O1*W*—H2*W*1⋯O3^i^	0.78 (3)	2.43 (3)	2.967 (2)	127 (3)
O5—H5⋯O1*W*^ii^	0.84	1.82	2.659 (3)	179
C7—H7*A*⋯O5	0.95	2.33	3.242 (3)	162

Symmetry codes: (i) 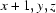; (ii) 
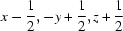
.

**Fig. 1.**
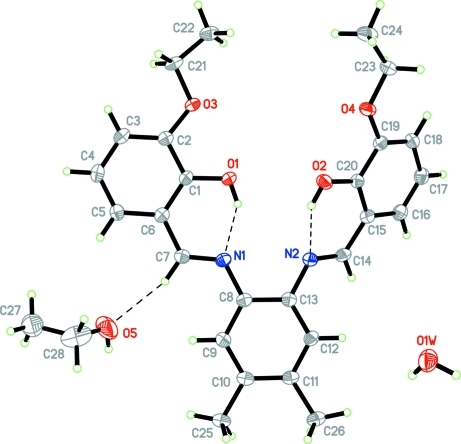
The asymmetric unit of the title compound, showing 50% probability displacement ellipsoids and the atomic numbering. Intra- and intermolecular hydrogen bonds are drawn as dashed lines.

## supplementary crystallographic information

### Comment

Schiff bases have received much attention because of their potential applications with some of these compounds exhibiting various pharmacological activities, such as anticancer (Dao *et al.*, 2000), anti-HIV (Sriram *et al.*, 2006), antibacterial and antifungal (Karthikeyan *et al.*, 2006) properties. Although numerous transition-metal complexes of Schiff bases have been structurally characterized (Granovski *et al.*, 1993), relatively few free Schiff bases have been similarly characterized. *N*-substituted salicylaldimines show photochromism and thermochromism in the solid state. These effects are produced by intramolecular proton transfer associated with a change in the π-electron configuration (Hadjioudis *et al.* 1987). In addition, some of them may be used as analytical reagents for the determination of trace elements (Eltayeb & Ahmed, 2005*a,b*) such as nickel in some natural food products (Fakhari *et al.*, 2005) or biologically important species (Shahrokhian *et al.*, 2000). As part of a general study of tetradenate and bidentate Schiff bases (Fun, Kargar & Kia 2008; Fun, Kia & Kargar 2008), we determined the structure of the title compound.

The asymmetric unit of the title compound, Fig. 1, comprises a Schiff base ligand, an ethanol and a water molecule of crystallization. All bonds lengths agree with standard values (Allen *et al.*,  1987). Strong intramolecular O—H···N hydrogen bonds generate *S(6)* ring motifs (Bernstein *et al.,* 1995). The hydrogen atoms of the water molecule make bifurcated intermolecular hydrogen bonds with the oxygen atoms of the hydroxyl and ethoxy groups with *R^2^_1_(5)* ring motifs (Bernstein *et al.,* 1995), which may, in part, influence the molecular configuration (Table 1). The dihedral angles between the central benzene ring and the two outer benzene rings of the Schiff base are 5.64 (8) and 44.78 (9)° which shows one of the outer benzene ring is twisted. The crystal structure is further stabilized by intermolecular C—H···O and π-π interactions [*Cg*1···*Cg*1^iii^ = 3.6139 (11) Å, (iii) x - 1/2, 1/2 - y, z - 1/2; *Cg*2···*Cg*2^iv^ = 3.7993 (11) Å, (iv) -x, -y, z - 1; *Cg*1 and *Cg*2 are the centroids of the C1–C6 and C15–C20 benzene rings].

### Experimental

The title compound was synthesized by adding 3-ethoxy-salicylaldehyde (4 mmol) to a solution of 4,5-dimethyl-*o*-phenylenediamine (2 mmol) in ethanol (20 ml). The mixture was refluxed with stirring for half an hour. The resultant yellow solution was filtered. Yellow single crystals of the title compound suitable for *X*-ray structure determination were recrystallized from ethanol by slow evaporation of the solvents at room temperature over several days.

### Refinement

H atoms of the hydroxy groups of the Schiff base and ethanol were positioned by a freely rotating O—H bond, see Table 1. The hydrogen of the water molecule were located from the difference Fourier map and refined freely. The remaining H atoms were positioned geometrically and refined as a riding model approximation. A rotating group model was used for the methyl groups.

### Crystal data

**Table:**

C_26_H_28_N_2_O_4_·C_2_H_6_O·H_2_O	*F*(000) = 1064
*M**_r_* = 496.59	*D*_x_ = 1.270 Mg m^−^^3^
Monoclinic, *P*2_1_/*n*	Mo *K*α radiation, λ = 0.71073 Å
Hall symbol: -P 2yn	Cell parameters from 4281 reflections
*a* = 9.5095 (5) Å	θ = 2.3–27.2°
*b* = 25.6633 (12) Å	µ = 0.09 mm^−^^1^
*c* = 10.7766 (5) Å	*T* = 100 K
β = 99.177 (2)°	Plate, orange
*V* = 2596.3 (2) Å^3^	0.45 × 0.12 × 0.02 mm
*Z* = 4	

### Data collection

**Table:**

Bruker SMART APEXII CCD area-detector diffractometer	7595 independent reflections
Radiation source: fine-focus sealed tube	4303 reflections with *I* > 2σ(*I*)
graphite	*R*_int_ = 0.068
φ and ω scans	θ_max_ = 30.1°, θ_min_ = 2.3°
Absorption correction: multi-scan (*SADABS*; Bruker, 2005)	*h* = −13→13
*T*_min_ = 0.961, *T*_max_ = 0.999	*k* = −36→32
32813 measured reflections	*l* = −14→15

### Refinement

**Table:**

Refinement on *F*^2^	Primary atom site location: structure-invariant direct methods
Least-squares matrix: full	Secondary atom site location: difference Fourier map
*R*[*F*^2^ > 2σ(*F*^2^)] = 0.066	Hydrogen site location: inferred from neighbouring sites
*wR*(*F*^2^) = 0.160	H atoms treated by a mixture of independent and constrained refinement
*S* = 1.03	*w* = 1/[σ^2^(*F*_o_^2^) + (0.0637*P*)^2^ + 0.5228*P*] where *P* = (*F*_o_^2^ + 2*F*_c_^2^)/3
7595 reflections	(Δ/σ)_max_ < 0.001
341 parameters	Δρ_max_ = 0.38 e Å^−^^3^
0 restraints	Δρ_min_ = −0.51 e Å^−^^3^

### Special details

**Table:**

Experimental. The crystal was placed in the cold stream of an Oxford Cyrosystems Cobra open-flow nitrogen cryostat (Cosier & Glazer, 1986) operating at 100.0 (1) K.
Geometry. All esds (except the esd in the dihedral angle between two l.s. planes) are estimated using the full covariance matrix. The cell esds are taken into account individually in the estimation of esds in distances, angles and torsion angles; correlations between esds in cell parameters are only used when they are defined by crystal symmetry. An approximate (isotropic) treatment of cell esds is used for estimating esds involving l.s. planes.
Refinement. Refinement of F^2^ against ALL reflections. The weighted R-factor wR and goodness of fit S are based on F^2^, conventional R-factors R are based on F, with F set to zero for negative F^2^. The threshold expression of F^2^ > 2sigma(F^2^) is used only for calculating R-factors(gt) etc. and is not relevant to the choice of reflections for refinement. R-factors based on F^2^ are statistically about twice as large as those based on F, and R- factors based on ALL data will be even larger.

### Fractional atomic coordinates and isotropic or equivalent isotropic displacement parameters (Å^2^)

**Table:**

	*x*	*y*	*z*	*U*_iso_*/*U*_eq_	
O1	−0.06040 (14)	0.16561 (5)	0.35336 (13)	0.0257 (3)	
H1	0.0253	0.1707	0.3841	0.039*	
O2	0.13555 (14)	0.07045 (5)	0.28857 (12)	0.0261 (3)	
H2	0.1782	0.0973	0.3193	0.039*	
O3	−0.33019 (13)	0.16242 (5)	0.25794 (12)	0.0250 (3)	
O4	−0.00004 (14)	−0.01584 (5)	0.22724 (12)	0.0276 (3)	
O5	0.1829 (2)	0.38301 (7)	0.43926 (18)	0.0696 (7)	
H5	0.2336	0.3938	0.5052	0.104*	
N1	0.16852 (16)	0.21898 (6)	0.42494 (13)	0.0202 (3)	
N2	0.30447 (16)	0.12535 (6)	0.45335 (14)	0.0221 (4)	
C1	−0.12615 (19)	0.21183 (7)	0.32673 (16)	0.0195 (4)	
C2	−0.2720 (2)	0.21128 (7)	0.27477 (17)	0.0212 (4)	
C3	−0.3428 (2)	0.25763 (8)	0.24528 (17)	0.0239 (4)	
H3A	−0.4410	0.2573	0.2103	0.029*	
C4	−0.2710 (2)	0.30517 (8)	0.26650 (18)	0.0271 (5)	
H4A	−0.3207	0.3369	0.2456	0.032*	
C5	−0.1292 (2)	0.30622 (8)	0.31737 (17)	0.0241 (4)	
H5A	−0.0812	0.3387	0.3319	0.029*	
C6	−0.0546 (2)	0.25953 (7)	0.34807 (16)	0.0197 (4)	
C7	0.0960 (2)	0.26133 (8)	0.39833 (16)	0.0216 (4)	
H7A	0.1422	0.2941	0.4120	0.026*	
C8	0.31619 (19)	0.21948 (7)	0.47186 (16)	0.0195 (4)	
C9	0.3962 (2)	0.26478 (8)	0.50515 (17)	0.0222 (4)	
H9A	0.3501	0.2977	0.4944	0.027*	
C10	0.5406 (2)	0.26294 (7)	0.55340 (16)	0.0212 (4)	
C11	0.6084 (2)	0.21406 (8)	0.56871 (17)	0.0216 (4)	
C12	0.5301 (2)	0.16936 (8)	0.53558 (16)	0.0222 (4)	
H12A	0.5768	0.1365	0.5447	0.027*	
C13	0.3845 (2)	0.17112 (7)	0.48914 (16)	0.0207 (4)	
C14	0.3273 (2)	0.08414 (7)	0.52055 (17)	0.0236 (4)	
H14A	0.3939	0.0857	0.5962	0.028*	
C15	0.2554 (2)	0.03506 (8)	0.48551 (17)	0.0229 (4)	
C16	0.2757 (2)	−0.00817 (8)	0.56692 (18)	0.0279 (5)	
H16A	0.3398	−0.0056	0.6439	0.033*	
C17	0.2041 (2)	−0.05394 (8)	0.53627 (19)	0.0287 (5)	
H17A	0.2191	−0.0829	0.5917	0.034*	
C18	0.1087 (2)	−0.05798 (8)	0.42326 (19)	0.0270 (4)	
H18A	0.0579	−0.0895	0.4029	0.032*	
C19	0.0884 (2)	−0.01644 (7)	0.34162 (17)	0.0232 (4)	
C20	0.16074 (19)	0.03062 (7)	0.37184 (17)	0.0208 (4)	
C21	−0.47745 (19)	0.15886 (8)	0.20175 (18)	0.0262 (4)	
H21A	−0.4913	0.1729	0.1151	0.031*	
H21B	−0.5377	0.1790	0.2515	0.031*	
C22	−0.5166 (2)	0.10202 (8)	0.20022 (19)	0.0310 (5)	
H22A	−0.6166	0.0978	0.1622	0.047*	
H22B	−0.5027	0.0887	0.2865	0.047*	
H22C	−0.4559	0.0826	0.1510	0.047*	
C23	−0.0850 (2)	−0.06146 (8)	0.1942 (2)	0.0305 (5)	
H23A	−0.1449	−0.0689	0.2592	0.037*	
H23B	−0.0231	−0.0920	0.1869	0.037*	
C24	−0.1771 (3)	−0.05042 (9)	0.0699 (2)	0.0410 (6)	
H24A	−0.2390	−0.0804	0.0451	0.061*	
H24B	−0.1166	−0.0441	0.0059	0.061*	
H24C	−0.2358	−0.0196	0.0778	0.061*	
C25	0.6220 (2)	0.31263 (8)	0.58803 (19)	0.0276 (5)	
H25A	0.5559	0.3422	0.5782	0.041*	
H25B	0.6935	0.3174	0.5328	0.041*	
H25C	0.6694	0.3106	0.6756	0.041*	
C26	0.7651 (2)	0.21015 (8)	0.62075 (18)	0.0279 (5)	
H26A	0.7960	0.1738	0.6173	0.042*	
H26B	0.7816	0.2222	0.7082	0.042*	
H26C	0.8195	0.2319	0.5705	0.042*	
C27	0.0836 (4)	0.45876 (12)	0.3369 (3)	0.0799 (11)	
H27A	0.0918	0.4816	0.2655	0.120*	
H27B	0.0995	0.4791	0.4148	0.120*	
H27C	−0.0118	0.4433	0.3258	0.120*	
C28	0.1892 (3)	0.41771 (13)	0.3437 (2)	0.0575 (8)	
H28A	0.1756	0.3986	0.2629	0.069*	
H28B	0.2852	0.4337	0.3553	0.069*	
O1W	0.84548 (19)	0.08448 (7)	0.14875 (16)	0.0412 (4)	
H1W1	0.920 (3)	0.0656 (11)	0.169 (3)	0.064 (9)*	
H2W1	0.855 (3)	0.1065 (13)	0.199 (3)	0.076 (11)*	

### Atomic displacement parameters (Å^2^)

**Table:**

	*U*^11^	*U*^22^	*U*^33^	*U*^12^	*U*^13^	*U*^23^
O1	0.0167 (6)	0.0236 (7)	0.0347 (8)	0.0002 (6)	−0.0026 (6)	0.0009 (6)
O2	0.0295 (8)	0.0200 (7)	0.0262 (7)	−0.0036 (6)	−0.0031 (6)	0.0021 (6)
O3	0.0155 (6)	0.0262 (8)	0.0318 (7)	0.0000 (6)	−0.0008 (5)	−0.0009 (6)
O4	0.0285 (7)	0.0219 (7)	0.0291 (7)	−0.0042 (6)	−0.0058 (6)	−0.0017 (6)
O5	0.0928 (16)	0.0370 (11)	0.0628 (13)	−0.0105 (10)	−0.0368 (11)	0.0001 (9)
N1	0.0185 (8)	0.0257 (9)	0.0163 (7)	−0.0007 (7)	0.0022 (6)	−0.0002 (6)
N2	0.0210 (8)	0.0214 (9)	0.0230 (8)	−0.0016 (7)	0.0012 (7)	−0.0028 (6)
C1	0.0194 (9)	0.0234 (10)	0.0158 (8)	0.0027 (8)	0.0030 (7)	−0.0003 (7)
C2	0.0195 (9)	0.0257 (11)	0.0185 (9)	0.0005 (8)	0.0035 (7)	−0.0015 (7)
C3	0.0197 (9)	0.0326 (11)	0.0191 (9)	0.0035 (8)	0.0027 (7)	0.0008 (8)
C4	0.0274 (11)	0.0256 (11)	0.0283 (10)	0.0083 (9)	0.0048 (8)	0.0031 (8)
C5	0.0267 (10)	0.0220 (10)	0.0240 (9)	0.0011 (8)	0.0054 (8)	−0.0004 (8)
C6	0.0208 (9)	0.0255 (10)	0.0136 (8)	0.0018 (8)	0.0046 (7)	−0.0007 (7)
C7	0.0229 (10)	0.0238 (10)	0.0182 (9)	−0.0029 (8)	0.0036 (7)	−0.0008 (7)
C8	0.0196 (9)	0.0248 (10)	0.0141 (8)	−0.0014 (8)	0.0030 (7)	−0.0007 (7)
C9	0.0248 (10)	0.0229 (10)	0.0192 (9)	−0.0010 (8)	0.0042 (8)	−0.0009 (7)
C10	0.0234 (9)	0.0262 (10)	0.0146 (8)	−0.0059 (8)	0.0050 (7)	−0.0012 (7)
C11	0.0190 (9)	0.0297 (11)	0.0172 (8)	−0.0036 (8)	0.0056 (7)	−0.0021 (8)
C12	0.0210 (9)	0.0255 (10)	0.0198 (9)	0.0018 (8)	0.0022 (7)	−0.0006 (8)
C13	0.0229 (9)	0.0233 (10)	0.0162 (8)	−0.0014 (8)	0.0039 (7)	−0.0020 (7)
C14	0.0196 (9)	0.0291 (11)	0.0211 (9)	−0.0006 (8)	−0.0005 (8)	−0.0018 (8)
C15	0.0198 (9)	0.0241 (10)	0.0243 (9)	−0.0004 (8)	0.0018 (8)	−0.0009 (8)
C16	0.0274 (10)	0.0277 (11)	0.0269 (10)	0.0032 (9)	−0.0005 (8)	0.0040 (8)
C17	0.0295 (11)	0.0252 (11)	0.0302 (10)	0.0032 (9)	0.0015 (9)	0.0052 (8)
C18	0.0255 (10)	0.0203 (10)	0.0348 (11)	−0.0004 (8)	0.0038 (9)	−0.0005 (8)
C19	0.0199 (9)	0.0239 (10)	0.0253 (9)	0.0014 (8)	0.0015 (8)	−0.0026 (8)
C20	0.0196 (9)	0.0191 (10)	0.0238 (9)	0.0010 (8)	0.0039 (8)	−0.0007 (7)
C21	0.0162 (9)	0.0386 (12)	0.0226 (9)	−0.0009 (9)	−0.0009 (7)	−0.0001 (8)
C22	0.0208 (10)	0.0402 (13)	0.0317 (11)	−0.0052 (9)	0.0030 (9)	−0.0031 (9)
C23	0.0278 (11)	0.0227 (11)	0.0389 (11)	−0.0052 (9)	−0.0009 (9)	−0.0054 (9)
C24	0.0407 (13)	0.0367 (13)	0.0400 (12)	−0.0085 (11)	−0.0106 (11)	−0.0076 (10)
C25	0.0259 (10)	0.0299 (11)	0.0268 (10)	−0.0085 (9)	0.0033 (8)	−0.0021 (8)
C26	0.0209 (10)	0.0366 (12)	0.0256 (10)	−0.0027 (9)	0.0024 (8)	−0.0043 (9)
C27	0.081 (2)	0.0466 (18)	0.093 (2)	−0.0021 (17)	−0.044 (2)	−0.0018 (16)
C28	0.0434 (15)	0.096 (2)	0.0340 (13)	−0.0122 (16)	0.0109 (12)	−0.0044 (14)
O1W	0.0394 (10)	0.0386 (10)	0.0406 (9)	0.0105 (8)	−0.0088 (8)	−0.0115 (8)

### Geometric parameters (Å, °)

**Table:**

O1—C1	1.350 (2)	C14—H14A	0.9500
O1—H1	0.8400	C15—C20	1.404 (2)
O2—C20	1.356 (2)	C15—C16	1.409 (3)
O2—H2	0.8400	C16—C17	1.372 (3)
O3—C2	1.371 (2)	C16—H16A	0.9500
O3—C21	1.437 (2)	C17—C18	1.401 (3)
O4—C19	1.377 (2)	C17—H17A	0.9500
O4—C23	1.435 (2)	C18—C19	1.376 (3)
O5—C28	1.370 (3)	C18—H18A	0.9500
O5—H5	0.8400	C19—C20	1.403 (3)
N1—C7	1.295 (2)	C21—C22	1.505 (3)
N1—C8	1.415 (2)	C21—H21A	0.9900
N2—C14	1.281 (2)	C21—H21B	0.9900
N2—C13	1.419 (2)	C22—H22A	0.9800
C1—C6	1.402 (3)	C22—H22B	0.9800
C1—C2	1.411 (2)	C22—H22C	0.9800
C2—C3	1.379 (3)	C23—C24	1.506 (3)
C3—C4	1.399 (3)	C23—H23A	0.9900
C3—H3A	0.9500	C23—H23B	0.9900
C4—C5	1.373 (3)	C24—H24A	0.9800
C4—H4A	0.9500	C24—H24B	0.9800
C5—C6	1.405 (3)	C24—H24C	0.9800
C5—H5A	0.9500	C25—H25A	0.9800
C6—C7	1.448 (3)	C25—H25B	0.9800
C7—H7A	0.9500	C25—H25C	0.9800
C8—C13	1.399 (3)	C26—H26A	0.9800
C8—C9	1.404 (3)	C26—H26B	0.9800
C9—C10	1.390 (3)	C26—H26C	0.9800
C9—H9A	0.9500	C27—C28	1.449 (4)
C10—C11	1.408 (3)	C27—H27A	0.9800
C10—C25	1.507 (3)	C27—H27B	0.9800
C11—C12	1.383 (3)	C27—H27C	0.9800
C11—C26	1.510 (3)	C28—H28A	0.9900
C12—C13	1.396 (3)	C28—H28B	0.9900
C12—H12A	0.9500	O1W—H1W1	0.86 (3)
C14—C15	1.454 (3)	O1W—H2W1	0.78 (3)
			
C1—O1—H1	109.5	C19—C18—C17	120.25 (18)
C20—O2—H2	109.5	C19—C18—H18A	119.9
C2—O3—C21	117.35 (14)	C17—C18—H18A	119.9
C19—O4—C23	116.90 (15)	C18—C19—O4	125.77 (17)
C28—O5—H5	109.5	C18—C19—C20	120.33 (17)
C7—N1—C8	122.35 (16)	O4—C19—C20	113.90 (16)
C14—N2—C13	119.64 (15)	O2—C20—C19	117.85 (16)
O1—C1—C6	122.41 (16)	O2—C20—C15	122.49 (17)
O1—C1—C2	117.93 (16)	C19—C20—C15	119.67 (17)
C6—C1—C2	119.66 (17)	O3—C21—C22	106.80 (16)
O3—C2—C3	125.94 (17)	O3—C21—H21A	110.4
O3—C2—C1	114.33 (16)	C22—C21—H21A	110.4
C3—C2—C1	119.73 (18)	O3—C21—H21B	110.4
C2—C3—C4	120.48 (17)	C22—C21—H21B	110.4
C2—C3—H3A	119.8	H21A—C21—H21B	108.6
C4—C3—H3A	119.8	C21—C22—H22A	109.5
C5—C4—C3	120.33 (18)	C21—C22—H22B	109.5
C5—C4—H4A	119.8	H22A—C22—H22B	109.5
C3—C4—H4A	119.8	C21—C22—H22C	109.5
C4—C5—C6	120.30 (18)	H22A—C22—H22C	109.5
C4—C5—H5A	119.9	H22B—C22—H22C	109.5
C6—C5—H5A	119.9	O4—C23—C24	107.00 (17)
C1—C6—C5	119.50 (17)	O4—C23—H23A	110.3
C1—C6—C7	120.96 (17)	C24—C23—H23A	110.3
C5—C6—C7	119.52 (17)	O4—C23—H23B	110.3
N1—C7—C6	121.07 (17)	C24—C23—H23B	110.3
N1—C7—H7A	119.5	H23A—C23—H23B	108.6
C6—C7—H7A	119.5	C23—C24—H24A	109.5
C13—C8—C9	118.63 (17)	C23—C24—H24B	109.5
C13—C8—N1	116.93 (16)	H24A—C24—H24B	109.5
C9—C8—N1	124.41 (17)	C23—C24—H24C	109.5
C10—C9—C8	122.02 (18)	H24A—C24—H24C	109.5
C10—C9—H9A	119.0	H24B—C24—H24C	109.5
C8—C9—H9A	119.0	C10—C25—H25A	109.5
C9—C10—C11	118.72 (17)	C10—C25—H25B	109.5
C9—C10—C25	120.09 (18)	H25A—C25—H25B	109.5
C11—C10—C25	121.19 (17)	C10—C25—H25C	109.5
C12—C11—C10	119.47 (17)	H25A—C25—H25C	109.5
C12—C11—C26	119.94 (18)	H25B—C25—H25C	109.5
C10—C11—C26	120.59 (17)	C11—C26—H26A	109.5
C11—C12—C13	121.84 (18)	C11—C26—H26B	109.5
C11—C12—H12A	119.1	H26A—C26—H26B	109.5
C13—C12—H12A	119.1	C11—C26—H26C	109.5
C12—C13—C8	119.30 (17)	H26A—C26—H26C	109.5
C12—C13—N2	121.93 (17)	H26B—C26—H26C	109.5
C8—C13—N2	118.71 (16)	C28—C27—H27A	109.5
N2—C14—C15	122.55 (17)	C28—C27—H27B	109.5
N2—C14—H14A	118.7	H27A—C27—H27B	109.5
C15—C14—H14A	118.7	C28—C27—H27C	109.5
C20—C15—C16	118.99 (17)	H27A—C27—H27C	109.5
C20—C15—C14	120.42 (17)	H27B—C27—H27C	109.5
C16—C15—C14	120.55 (17)	O5—C28—C27	113.5 (3)
C17—C16—C15	120.73 (18)	O5—C28—H28A	108.9
C17—C16—H16A	119.6	C27—C28—H28A	108.9
C15—C16—H16A	119.6	O5—C28—H28B	108.9
C16—C17—C18	120.03 (18)	C27—C28—H28B	108.9
C16—C17—H17A	120.0	H28A—C28—H28B	107.7
C18—C17—H17A	120.0	H1W1—O1W—H2W1	103 (3)
			
C21—O3—C2—C3	1.6 (3)	C26—C11—C12—C13	178.89 (17)
C21—O3—C2—C1	−178.13 (16)	C11—C12—C13—C8	2.0 (3)
O1—C1—C2—O3	0.3 (2)	C11—C12—C13—N2	179.06 (17)
C6—C1—C2—O3	179.82 (16)	C9—C8—C13—C12	−1.7 (3)
O1—C1—C2—C3	−179.47 (16)	N1—C8—C13—C12	−179.82 (16)
C6—C1—C2—C3	0.1 (3)	C9—C8—C13—N2	−178.84 (16)
O3—C2—C3—C4	−179.69 (17)	N1—C8—C13—N2	3.0 (2)
C1—C2—C3—C4	0.0 (3)	C14—N2—C13—C12	40.6 (3)
C2—C3—C4—C5	−0.2 (3)	C14—N2—C13—C8	−142.33 (18)
C3—C4—C5—C6	0.3 (3)	C13—N2—C14—C15	−176.25 (17)
O1—C1—C6—C5	179.54 (16)	N2—C14—C15—C20	2.6 (3)
C2—C1—C6—C5	0.0 (3)	N2—C14—C15—C16	−175.16 (19)
O1—C1—C6—C7	1.1 (3)	C20—C15—C16—C17	−0.2 (3)
C2—C1—C6—C7	−178.44 (17)	C14—C15—C16—C17	177.60 (19)
C4—C5—C6—C1	−0.2 (3)	C15—C16—C17—C18	−0.4 (3)
C4—C5—C6—C7	178.27 (18)	C16—C17—C18—C19	1.1 (3)
C8—N1—C7—C6	178.76 (16)	C17—C18—C19—O4	178.80 (19)
C1—C6—C7—N1	0.0 (3)	C17—C18—C19—C20	−1.2 (3)
C5—C6—C7—N1	−178.43 (17)	C23—O4—C19—C18	4.2 (3)
C7—N1—C8—C13	−175.89 (16)	C23—O4—C19—C20	−175.79 (17)
C7—N1—C8—C9	6.1 (3)	C18—C19—C20—O2	−179.31 (18)
C13—C8—C9—C10	0.6 (3)	O4—C19—C20—O2	0.6 (2)
N1—C8—C9—C10	178.60 (17)	C18—C19—C20—C15	0.6 (3)
C8—C9—C10—C11	0.2 (3)	O4—C19—C20—C15	−179.42 (17)
C8—C9—C10—C25	−179.88 (17)	C16—C15—C20—O2	−179.95 (18)
C9—C10—C11—C12	0.0 (3)	C14—C15—C20—O2	2.2 (3)
C25—C10—C11—C12	−179.86 (17)	C16—C15—C20—C19	0.1 (3)
C9—C10—C11—C26	179.99 (17)	C14—C15—C20—C19	−177.71 (18)
C25—C10—C11—C26	0.1 (3)	C2—O3—C21—C22	−177.08 (16)
C10—C11—C12—C13	−1.2 (3)	C19—O4—C23—C24	177.06 (17)

### Hydrogen-bond geometry (Å, °)

**Table:**

*D*—H···*A*	*D*—H	H···*A*	*D*···*A*	*D*—H···*A*
O1—H1···N1	0.84	1.84	2.584 (2)	146
O1W—H1W1···O2^i^	0.86 (3)	2.24 (3)	2.947 (2)	139 (3)
O1W—H1W1···O4^i^	0.86 (3)	2.28 (3)	3.018 (2)	145 (3)
O2—H2···N2	0.84	1.87	2.609 (2)	146
O1W—H2W1···O1^i^	0.78 (3)	2.30 (3)	3.061 (2)	166 (3)
O1W—H2W1···O3^i^	0.78 (3)	2.43 (3)	2.967 (2)	127 (3)
O5—H5···O1W^ii^	0.84	1.82	2.659 (3)	179
C7—H7A···O5	0.95	2.33	3.242 (3)	162

Symmetry codes: (i) *x*+1, *y*, *z*; (ii) *x*−1/2, −*y*+1/2, *z*+1/2.
